# Evaluation of electrolyte element composition in human tissue by laser-induced breakdown spectroscopy (LIBS)

**DOI:** 10.1038/s41598-022-20825-0

**Published:** 2022-09-30

**Authors:** Philipp Winnand, K. Olaf Boernsen, Georgi Bodurov, Matthias Lammert, Frank Hölzle, Ali Modabber

**Affiliations:** 1 Department of Oral and Maxillofacial Surgery, University Hospital RWTH Aachen, Pauwelsstraße 30, 52074 Aachen, Germany; 2 Advanced Osteotomy Tools AG, Wallstraße 6, 4051 Basel, Switzerland; 3 Institute of Pathology, University Hospital RWTH Aachen, Pauwelsstraße 30, 52074 Aachen, Germany

**Keywords:** Solid-state lasers, Laser-produced plasmas, Diagnostic markers, Characterization and analytical techniques, Optical spectroscopy, Tissues, Optical spectroscopy, Optics and photonics, Applied optics

## Abstract

Laser-induced breakdown spectroscopy (LIBS) enables the direct measurement of cell electrolyte concentrations. The utility of LIBS spectra in biomarker studies is limited because these studies rarely consider basic physical principles. The aim of this study was to test the suitability of LIBS spectra as an analytical method for biomarker assays and to evaluate the composition of electrolyte elements in human biomaterial. LIBS as an analytical method was evaluated by establishing KCl calibration curves to demonstrate linearity, by the correct identification of emission lines with corresponding reference spectra, and by the feasibility to use LIBS in human biomaterial, analyzing striated muscle tissues from the oral regions of two patients. Lorentzian peak fit and peak area calculations resulted in better linearity and reduced shot-to-shot variance. Correct quantitative measurement allowed for differentiation of human biomaterial between patients, and determination of the concentration ratios of main electrolytes within human tissue. The clinical significance of LIBS spectra should be evaluated using peak area rather than peak intensity. LIBS might be a promising tool for analyzing a small group of living cells. Due to linearity, specificity and robustness of the proposed analytical method, LIBS could be a component of future biomarker studies.

## Introduction

Electrolyte hemostasis of calcium (Ca), potassium (K), and sodium (Na) is strictly regulated. A change in electrolyte balance can have a primary cause, such as recurrent vomiting, diarrhea, or high fever, or can be a result of a general disease, tumor, or tumor-associated side effect^1^. Changes in the extracellular space are part of routine laboratory analysis and are therefore easy to detect. The situation is different with the concentration of trace elements in living biological tissues, as well as within the cells themselves. Electrolyte balance is essential for the functionality of tissue cells^2^, while the regulation and dynamics of electrolyte imbalance are key contributors to tumorigenic processes^3^. However, no analytical method exists that can easily detect these changes within corresponding cells. Raman spectroscopy was introduced as a potential tool for real-time tissue diagnostics by evaluating the molecular water spectrum^4,5^. Instruments based on mass spectrometric molecular analysis are complex and only allow for the indirect evaluation of cellular processes^6^. At present, indirect and direct measurement techniques are frequently combined to provide even much more information about a biological sample. In this context, laser-induced breakdown spectroscopy (LIBS) is frequently used as a direct measurement technique for hybrid systems^7,8^.

LIBS measures the atomic emission spectra of elements directly from laser-produced hot plasma. Despite the possibility of simulating plasma using Saha-LTE (local thermodynamic equilibrium) approximations^9,10^, the inhomogeneous nature of biomaterial heavily interferes with the predictable absorption and conversion kinematics of photon energy. Therefore, the temperature within the plasma, the lattice vibration in a solid-state body, and the density and liquid content of the sample under investigation determine the conditions of the plasma plume. Under the harsh temperature conditions of plasma formation and the atomization of minimal tissue surfaces, the ablated biological material is degraded into its atomic elements in a multitude of different chemical reactions, with partially incomplete molecular decomposition occurring^11^. For human tissues, these thermal processes mean energy absorption by peptides, proteins, lipids, and other molecules. However, water and low photon density are constant hurdles for LIBS-based analysis, as they dramatically quench light emission and the LIBS signal^12,13^.

As a result of these processes, the presence of a high number of excitation levels and ground states leads to an element-specific number of emission lines. Based on this characteristic emission spectrum, each element can be identified and quantified using optical detection and spectroscopic analysis methods^14^. In addition to these methodological specifications, possible pitfalls in the evaluation of LIBS spectra must be considered, especially when only the peak intensities of the LIBS signals are calculated. At high concentrations, self-absorption leads to a reduction in peak intensity, with a simultaneous broadening of peak width. Under the same conditions, atoms with low abundance show low intensities with narrow peak widths. This form of self-broadening, also known as the Stark effect, leads to peak splitting and nonlinear calibration curves^15^. Since the LIBS emission peaks follow a Lorentzian distribution, different peak shapes result, depending on the signal intensity. Therefore, it is impossible to draw reliable conclusions regarding increases and decreases in LIBS signals from the comparison between low- and high-intensity peaks^16,17^. These shortcomings pose major challenges in clinical biomarker studies that use LIBS spectra and thus limit the reliability of analytical, quantitative assays, which should be used in clinical biomarker assays^18^.

Although LIBS-based analysis of atomic emission spectra and the distribution of elements has been used to distinguish between different tissue types^19^ and between tumor and healthy control tissue^20^, current biomarker studies often do not consider the importance of peak area calculation or the influence of the Stark effect on the biological significance of LIBS spectra.

Linearity, specificity, and robustness are crucial not only for validated biomarker assays^21,22^, but also for the use of LIBS in clinical studies. This work aims to evaluate an analytical method that can potentially meet these requirements. On this basis, this study will investigate whether LIBS can be a suitable tool for biomarker studies by testing the linearity of the calibration curve and by identifying the electrolyte emission lines with the corresponding reference spectra. Furthermore, LIBS will be validated as a direct measurement technique on human biomaterial to determine tissue electrolyte composition in real time. Due to the numerous challenges with native human samples, this paper focuses exclusively on LIBS. This could pave the way for the successful implementation of multimodal approaches in combination with LIBS.

## Materials and methods

### Ethical approval

Ethical approval was given by the Ethics Committee of the Medical Faculty of RWTH Aachen, Germany (EK 392/21). Written informed consent was obtained from all participants prior to the surgical procedure. Striated muscle tissues originated from the oral regions of two patients and had dimensions of 8 × 5 × 2 mm. Human biomaterial was collected in cooperation with the RWTH centralized Biomaterial Bank (RWTH cBMB) of the Medical Faculty of RWTH Aachen University. This trial was performed in accordance with the current version of the Declaration of Helsinki.

### Experimental setup

The technical setup was typical of LIBS instrumentation. The sample was placed in a sample holder mounted on a manual, two-dimensional stage. Laser intensity, focusing lens, and photon density are important parameters for human tissue analysis. All components were designed for the optimal measurement of the native tissue samples.

The measurements were performed with the AOT LIBS System (Advanced Osteotomy Tools AG, Basel, Switzerland). Briefly, the beam of a short laser pulse (τ = 8 ns) of a Q-switched Nd:YAG laser (λ = 1064 nm; frequency = 10 Hz) was focused on the target surface, where an area approximately 240 μm in diameter was covered. The radiation of the laser-produced plasma was collected by an optical system and forwarded via an optical fiber to a spectrometer covering a range of 127–868 nm.

All samples used in the work were measured under identical setup and laser conditions. Only two laser shots were accumulated for all calculations. More technical details of the experimental setup will be published elsewhere.

### Pattern recognition and detected lines in human tissues

The light emissions of the elements and their corresponding wavelengths in LIBS experiments depend primarily on the concentration of the main electrolytes in the sample. However, the number of analytically accessible elements in tissue samples is limited, and a wide range of wavelengths is often published for particular elements.

To identify all the line patterns of a specific element in an unknown tissue sample, reference spectra were recorded with pure salt solutions for all main electrolytes. The intensities of a LIBS spectrum depend on the calibration of the spectrometer, the optical components, their coatings, and their individual transition rates. By using reference spectra, experiment-specific variables can be excluded. Consequently, elementary emission lines are available that are specific to the laser power and the spectrometer used.

The spectrum of a particular element can also be simulated using the National Institute of Standards and Technology (NIST) database^23^. Using the reference spectra and the simulation, the peak spectra of the elements can be identified in detail. In this study, human muscle tissue was used to observe, identify, and detect the following elements^14^: Carbon (C), cyanide (CN), iron (Fe), magnesium (Mg), Ca, calcium oxide (CaO), calcium hydroxide (CaOH), barium (Ba), strontium (Sr), hydrogen (H), Na, K, oxygen (O), and nitrogen (N).

### Calibration curve and quantitative analysis of reference spectra

For the calibration and reference experiments, ash-free round filter types (Binzer and Munktell Filter, Battenberg, Germany) with a diameter of 70 mm (Qual. 4) were used. Generally, these ash-free filters are used in wet quantitative chemical analysis. After combustion, the ash content is less than 0.01%. This material is ideally suited as a LIBS sample carrier, as it is free of any metals in the form of salts or oxides. Therefore, a blank filter shows absolutely no emission lines of metal atoms. For the calibration curve, nine samples were prepared between 1 and 144 nmol/mm^2^ KCl. All samples were spiked with a fixed amount of NaCl. The calibration curve of KCl was normalized to the Na signal, which was used as the internal standard.

To calculate the peak areas, the measured peak was fitted with a Lorentzian function. The Lorentzian parameter determines the peak intensity, the full width at half maximum (FWHM), and the exact emission wavelength, all of which were used to calculate the peak area. Each data point contained two laser shots. The K peak area was the sum of the two peak areas of the two main K emission lines at 766.39 nm and 769.98 nm.

To prepare the reference spectra, the filter paper was completely soaked with a 1 m salt solution until the entire filter was wet but had no water droplets. The filter was then air-dried overnight. Salt loading was determined by weighing the filter before and after the loading. The unit of nmol/mm^2^ was chosen for two reasons. First, the signal intensity of a given element increases in proportion to the diameter of the laser beam and the volume examined on the target material. Second, filter paper bears the risk of forming gradients with higher salt concentrations on its surface because the crystallization and drying processes occur on the surface only.

On average, 8 laser shots with good signal responses could be acquired on a single laser spot. The loaded total amount of salt per mm^2^ was divided by 8 to get an idea of the salt consumption of a single laser shot. However, this was only an assumption that was as close as possible to reality. The LIBS spectrum recorded on clean filter paper (without salts) showed no element peaks under the conditions used. Therefore, subtraction of the background spectrum was omitted.

### LIBS-based analysis of muscle tissue

First, a macroscopic evaluation of the muscle tissue specimens was performed by a surgeon and a pathologist. The specimens were stored at − 20 °C until the LIBS experiments were performed. The tissue specimens were rinsed with NaCl (0.9%) and carefully blotted dry with gauze, 60 min prior to the LIBS experiments. The muscle tissue was natively lasered. For each LIBS experiment, spectra were collected from 40 spots, using 30 shots per spot. Thirty shots were chosen to allow for a better shot-to-shot comparison, if needed. The LIBS experiments were each performed within 30 min. According to the histopathological routine, the muscle tissue specimens were fixed in formalin, embedded in paraffin, cut, and H&E-stained. The tissue areas of the specimens were histologically validated.

### Calculation of Na/K ratios in human biomaterial samples

The peak areas of the Na (589.18 nm and 819.22 nm) and K (766.39 nm and 769.98 nm) emission lines were used to determine the Na/K ratio within the muscle tissues of the two patients. In this way, an analysis of the first 4 laser shots was performed with respect to their Na/K ratios. The results were compared with the Na/K ratios calculated from the blood sera of the respective patients from the hospital’s internal database, which were taken immediately before surgery.

## Results

### Quantification of the self-broadening Stark effect

**Figure 1:**
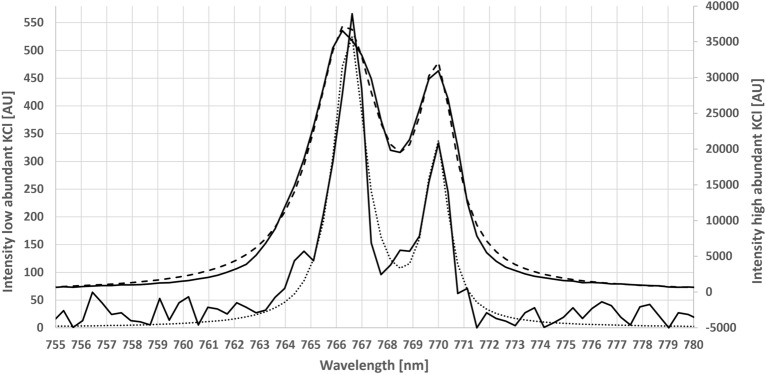
Comparison of two KCl concentrations (1 and 144 nmol/mm^2^) on filter paper. Self-broadening of abundant signals leads to a dramatic increase in peak width and a loss of intensity. The y-axis for the area of the high concentration peak is on the right side, while the y-axis for the low concentration is on the left side. The dotted lines show the fitted curves, while the solid lines represent the measured spectra. The broader peaks represent the situation at 144 nmol/mm^2^ and the narrow peaks are measured at 1 nmol/mm^2^.

The calibration curve was tested with K and Na according to internal standards. An initial approach simply using the intensities of a K and Na peak showed limited linearity, but a curved shape at higher K concentrations^24–27^. Figure 1 shows an overlay with low and high K concentrations and makes the effect of peak broadening visible by giving each concentration its own y-axis. Consideration of the Stark effect illustrates that there was no linearity when using peak intensity only. To quantify this peak broadening effect, the peaks were fitted with a Lorentzian curve. This curve (dashed lines in Fig. 1) represents the average of all measured data points over a given peak.

### The influence of peak area calculation on the linearity of the K calibration curve

**Figure 2:**
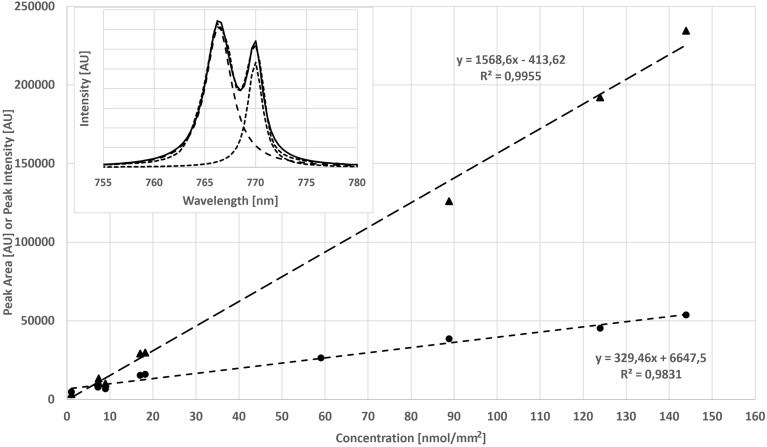
Calibration curves of K (KCl) normalized to NaCl on saturated ash-free filter paper as Lorentzian peak fitting area (triangle) and as peak intensity (circle). Signal intensity was measured in arbitrary units (AU) as the Lorentzian peak fitting area or as peak intensity. The embedded figure shows the two single Lorentzian peak fits for the two K emission lines at 766.36 nm and 769.95 nm (calculated from the Lorentzian peak) from 144 nmol/mm^2^ KCl on filter paper. The solid line corresponds to the result that was measured, and the dotted lines show the two individual fits.

An accurate K calibration curve is challenged by the characteristic existence of two overlapping peaks with broad peak bases. Individual peak fitting allowed us to obtain two fully separated peaks for further calculations^28^, as shown in the embedded plot of Fig. 2. Each peak area was calculated from the exact peak position, the peak width, and the Lorentzian function, and accurately reflected the reality of the peak in the LIBS experiments. However, determining the correct K peak for the best linear calibration curve is challenging because each K atomic nucleus can return its electron to the ground state through these two transitions, with light emissions at 766.39 nm and 769.98 nm. Conversely, each photon detected represents a K nucleus. Thus, all emission lines of a given element should be used if quantitative information is needed.

To prove this, a new calibration curve was constructed using the peak areas of the two main K emission lines, as shown in Fig. 2. High linearity (R^2^ = 0.9955) over the entire concentration range was achieved by using the peak area approach. In contrast, the calibration curve for the peak intensity showed good linearity only above 15 nmol/mm^2^. However, below 15 nmol/mm^2^, the calibration curve was not as well fitted as before. In this context, it must be noted that the intercept for the peak intensity calibration has increased to 6647, while the intercept for the peak area was only 413 with good linearity over the whole concentration range. Both calibration curves were normalized with the Na emission lines at 589.21 nm. However, it should be noted that very similar calibration curves were observed for both data sets, when no normalization was applied.

### Inter-patient comparison of LIBS spectra from muscle tissue

**Figure 3:**
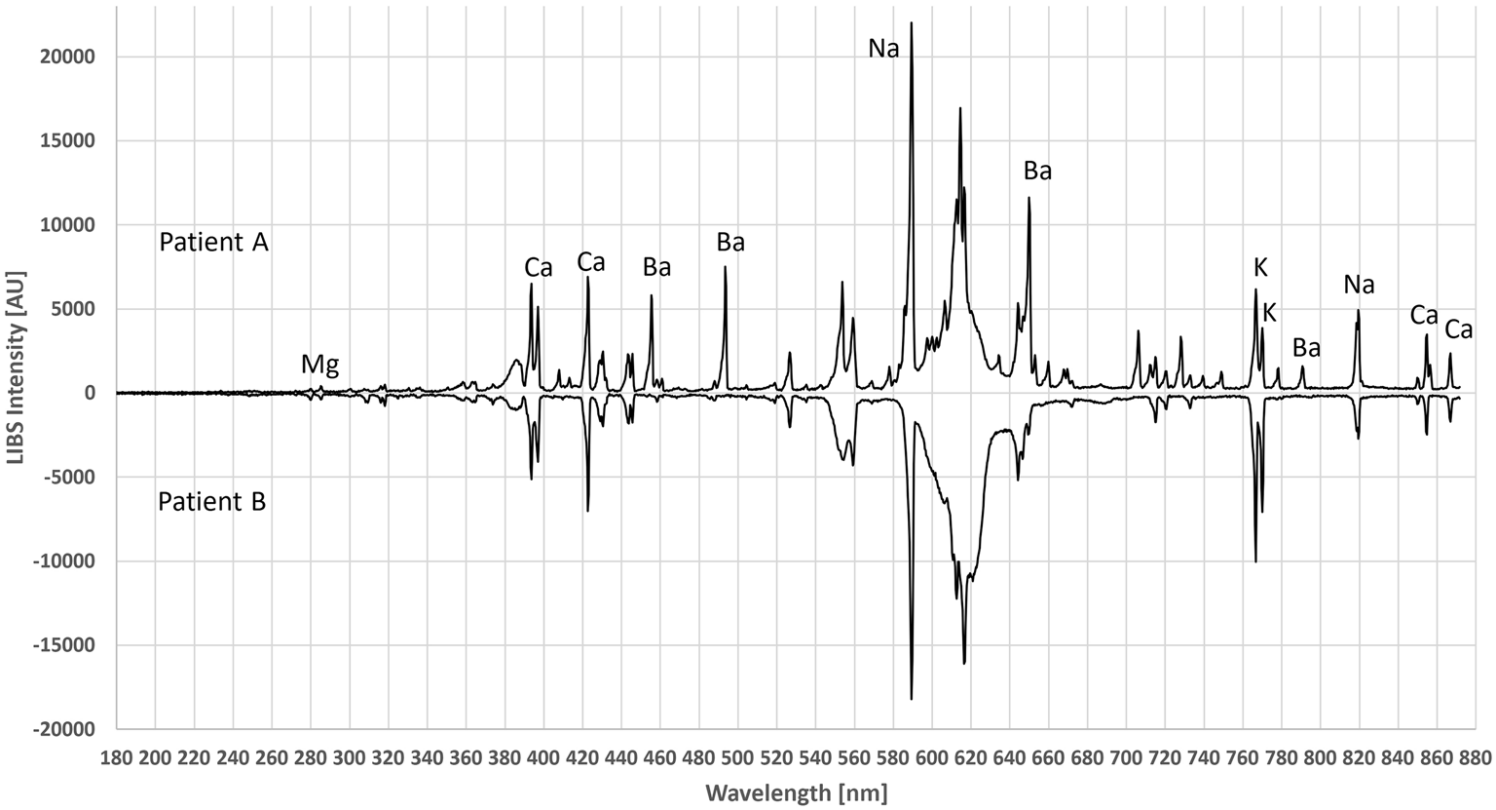
Comparison of the LIBS spectra of the two patients. Interestingly, the muscle of Patient A shows high Ba concentration—a very rare case—while Patient B shows a more typical spectrum of human muscle cells.

For further evaluation, muscle tissues from two patients were compared to evaluate LIBS. Figure 3 shows the typical single-shot LIBS spectra of the two patients without any further data processing. The main elements identified were Ca, Na, and K, while Patient A also had a number of Ba emission lines.

### Quantification of muscle tissue and comparison to the reference spectra of CaCl_2_ and BaCl_2_

**Figure 4:**
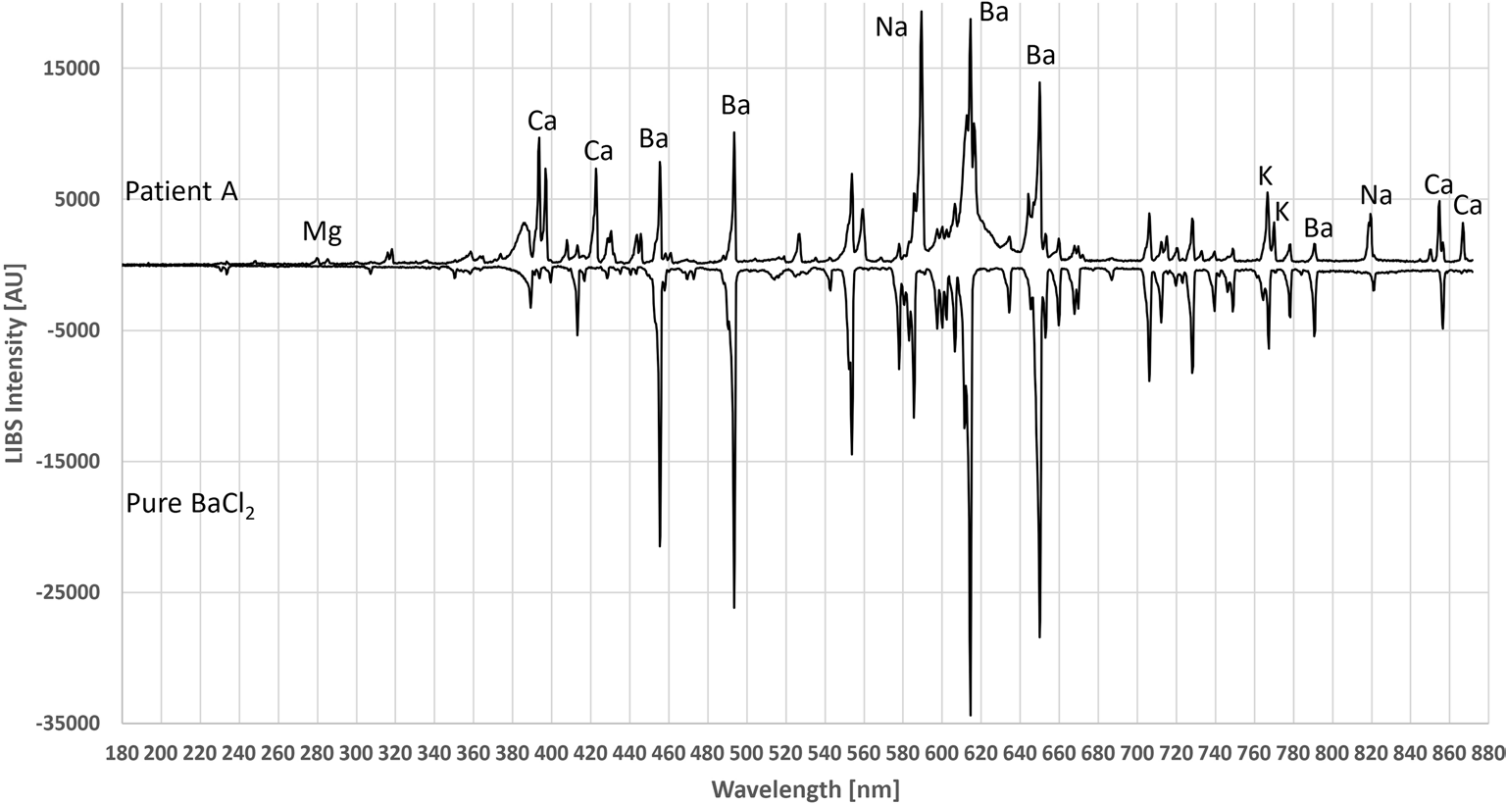
Comparison between the LIBS spectrum of Patient A and BaCl_2_ reference on filter paper. The emission lines from chlorine are not visible under current conditions.

**Figure 5:**
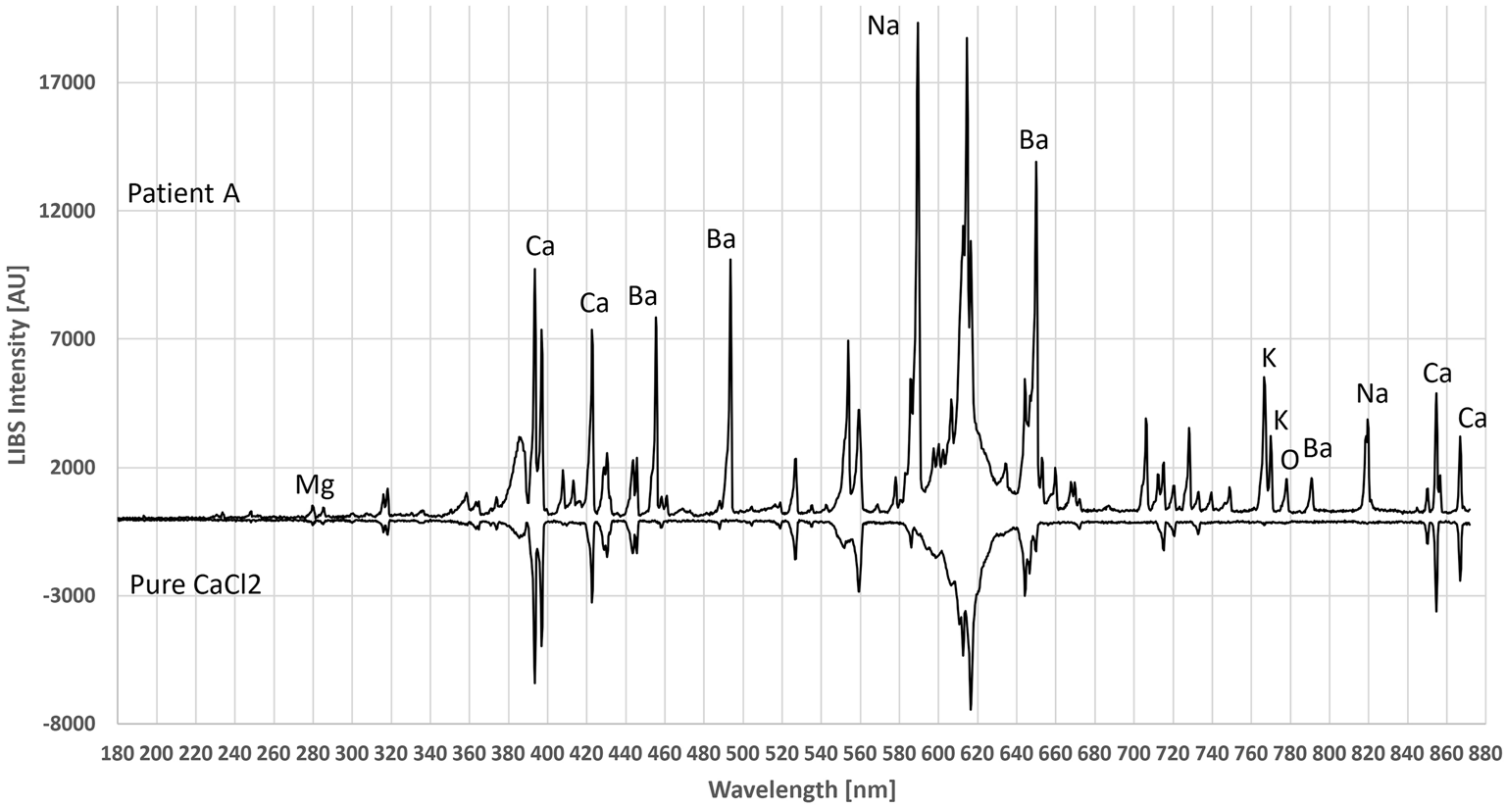
Comparison between the LIBS spectrum of Patient A with CaCl_2_ reference on filter paper. The emission lines from chlorine are not visible under current conditions.

Identifying all visible emission peaks from the literature is time consuming and difficult due to differences in intensity and wavelength. Therefore, reference samples were taken for the main components. Due to the identical experimental conditions, wavelengths, peak shapes, and the number of emission lines of pure salt solutions were directly compared to the human tissue samples. Since the ash-free filter paper used for quantitative chemical analysis did not contain any oxides, metals or salts, there was no noticeable background. Figure 4 compares Patient A’s spectrum to the reference spectrum of BaCl_2_. The same was done with CaCl_2_ in Fig. 5. However, there were also a number of CaO and CaOH (radical) lines, recognized by their broader peak shapes, in the areas of 546–554 nm and 600–630 nm^29,30^. Due to the 40,000 times higher LOD compared to the Ca^14^, no chloride emission lines were visible. It should be noted that calcium chloride and barium chloride are crystallized with hydrate water to CaCl_2_∙2H_2_O and BaCl_2_∙2H_2_O, respectively, which were used in the applied calculations.

### Determination of electrolyte composition with Na/K ratios

**Figure 6:**
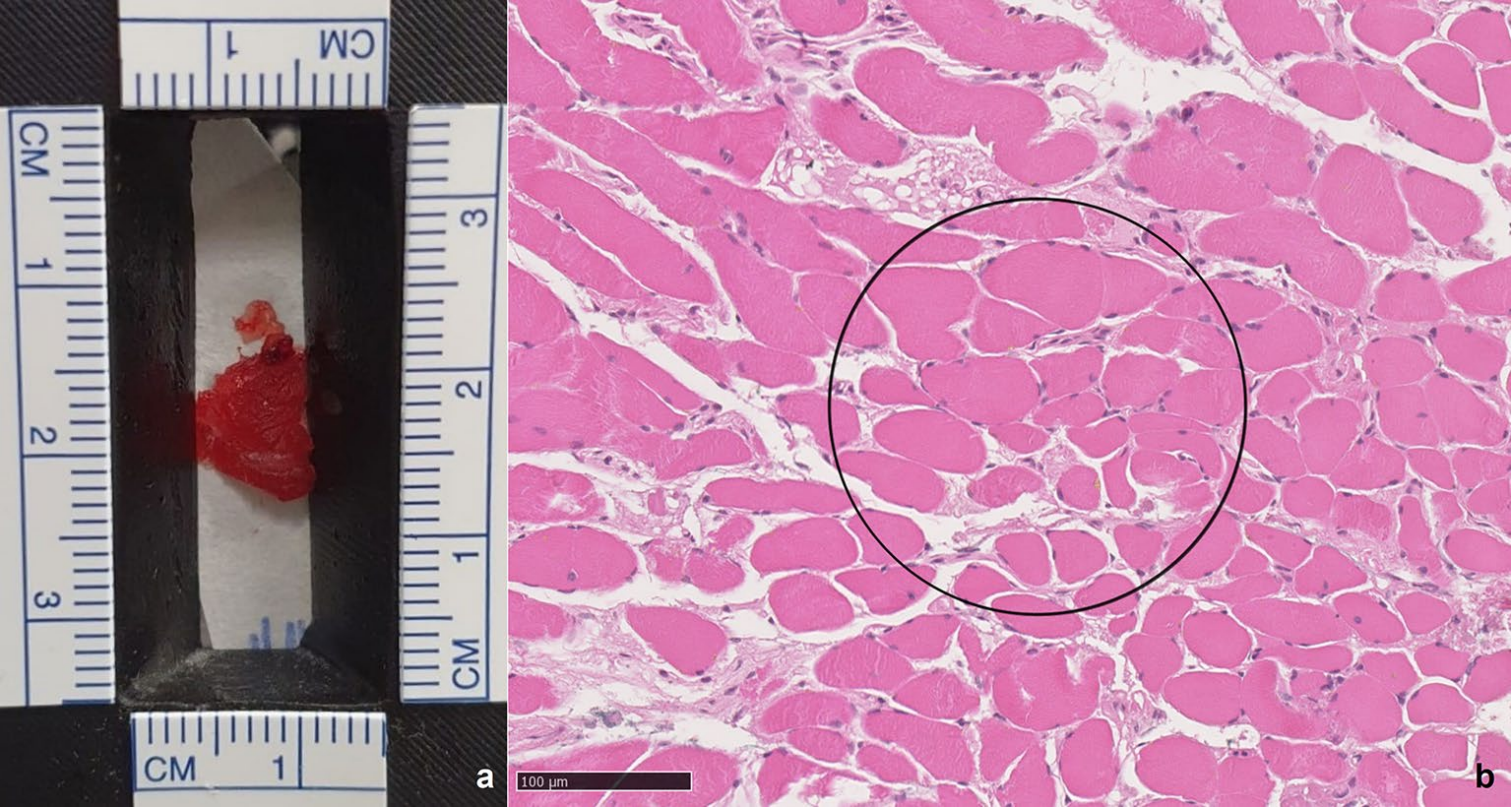
Macroscopic (**a**) and histological (**b**) view (10 × magnification) of striated muscle tissue. The circle illustrates the area of a typical laser shot (240 μm) and the relationship between the cells and the intercellular space.

Another way to compare data, even across different platforms, is to use the ratios of two elements. For the two patients in Fig. 3, a Na/K ratio was calculated using an amplification peak area across the two combined emission lines (see above). Table 1 shows the Na/K ratio for the two patients from the muscle tissue studied with LIBS, from the corresponding patients’ sera found in the clinical data, and from the literature. Figure 6 shows the histological view of the muscle cells, with an average size of 20–40 μm. The circle covers the area of a typical laser shot and illustrates the relationship between the cells and the intercellular space. Due to the size of the laser spot cylinder (240 μm diameter and 0.5 μm thickness), the LIBS signals were acquired from both the cells and the intercellular space. Therefore, the Na/K ratios represent a mixture of the Na and K content of both compartments.

**Table 1:** Comparison of Na/K ratios in the blood and muscle tissues of two corresponding patients.

	Serum Na/K	LIBS spot Peak area Na/K K intensity corrected*	+ /− SD	Cell intra^a^	Cell inter^a^
Patient A	34.25	2.18	0.0456		
Patient B	31.30	1.94	0.399		
Range^b^	25–38			0.143	36

^a^K peak area was corrected by a factor of 1.719. In this way, the same amounts of Na and K led to the same peak area.

^b^From the literature.

## Discussion

The LIBS process itself is based on a number of different processes that all occur in parallel or sequentially in time. Knowledge of these interdependent steps (Table 2) is important for understanding and interpreting signal behavior in LIBS experiments on biological tissues^11^. In this study, the peak area calculation was evaluated using the LIBS spectra of human muscle tissues.

**Table 2:** Basic steps and processes in laser-induced breakdown spectroscopy.

	Main Steps in LIBS	Remarks
1	Laser pulse (fs or ns; wavelength; power)	Constant photon energy pulse
2	Light absorption by target material	Photon energy transfer to heat, depending on material properties and water content
		Formation of plasma shock wave^58,59^
		Continuum emissions up to 1000 ns after the laser pulse^59^
		Inhomogeneous plasma with hot and cold areas^58,59^
		Light emission of atoms (and stable molecules) 1–15 μs after the laser pulse^59^
		Plasma density and plasma temperature in equilibrium
3	Material ablation and plasma formation	Breakdown of molecules and clusters
		Incomplete molecule breakdown and formation of CN, C2, CaO, etc.^11^
		Chemical reactions with ambient air^11^
		Emission of element-specific wavelengths
		Plasma density and plasma temperature control the energy distribution in the excited S1 state and thus the transition pathways to the ground state
4	Plasma plume expansion	Self-broadening of emitting light peaks (Stark effect)
		Cold atoms of the same light-emitting element absorb their photons. Formation of a “dip” in the center of the peak, which has a Lorentzian distribution
5	Collection of emitted light within a time gate	Delay time and integration time, nearly independent of daylight. Light collection optics focused on the plasma plume
6	Qualitative view	Identification of an element by its emitting wavelengths. Intensity of the individual peaks, depending on the LIBS setup and hardware. Generated identical spectra for a given element by measuring the reference spectra with the same hardware and settings
7	Quantitative view	Presentation of most elements with all their major emitting wavelengths. Peak area and Lorentzian fit are the best options for quantitative aspects^59,60^

LIBS has long been a successful analytical method used in the study of metals, alloys, and geological rocks^31–33^. Here, the conditions are much more controllable because the nature of the solid material makes the variations in the LIBS process small. However, the target material in biological tissues is much more complex, resulting in highly variable and rarely reproducible conditions for the plasma formation^11^. In previous LIBS experiments, these obstacles were addressed by averaging up to 100 laser shots^34^. However, this method does not reflect the complexity of the emission spectra of certain elements and does not provide reliable quantitative information^18^, especially if the elements are to be used as biomarkers of clinical significance. Recently, multimodal systems were proposed to characterize biological tissues. In this context, the combination of LIBS with Raman spectroscopy successfully identified the atomic and molecular composition of complex biological samples like renal-calculi^8^. Great efforts are also being made to develop combined methods such as coherent anti-Stokes Raman scattering for tumor detection^35,36^. Although the combination of direct and indirect analytical methods is highly reasonable, the difficulty of analyzing native, soft-tissue biomaterials is rarely considered. The current work addresses this problem using the specific application of LIBS as an example for other spectroscopic analysis methods. Moreover, all the methods described are limited to the analysis of surfaces only. In contrast, LIBS can also be used under difficult or extreme conditions, such as under water^37^.

To demonstrate the difficulties in controlling LIBS experiments and evaluate the impact of the analysis of clinical samples, a simple calibration experiment with low and high K concentrations was performed. As shown with the KCl calibration sample on filter paper (Fig. 2), consideration of peak shape transition pathways generates reproducible and more solid results. Using simple peak intensities resulted in a high intercept and a poor fitting at the lower end of the calibration curve, so a curved calibration curve could result in a better fit. Another option would be to work with two linear calibration curves, one for the lower end and the other one for the upper end. However, both methods are not satisfactory. This curved calibration curve is attributed to the Stark effect, which suppresses the peak intensities at higher concentrations and simultaneously leads to peak broadening. In very simplified terms, no emitted photons are lost due to the Stark effect, since the emitted photons merely shift their wavelengths and are all detected in the end^15,38^. As the presented approach shows, only the peak area can serve as a good approximation to compensate for the intensity loss. The fact that similar results were observed when no normalizations were applied indicates that the sample preparation was reproducible for all concentrations and that the sample was evenly distributed on the filter paper. It is also an indication that the LIBS system used for this study works very homogeneously. In this context, peak fitting offers the possibility of separating overlapping peak shapes, which allows for an accurate calculation of the individual peaks^39^. A fit for a given peak is always performed with 10–50 measured data points following the peak shape. Therefore, the peak area calculation captures the signal behavior of the elements much more accurately than if only a single (highest) data point were evaluated. In addition, the background intensities (scattering) or other effects can be easily excluded by the fitted peak shape. If this approach is followed systematically, shot-to-shot variability can be reduced. This means that as few as two laser shots are sufficient to generate reproducible data. Although modern machine learning algorithms or neural networks can be used just as efficiently to develop a calibration curve^40,41^, the KCl calibration sample provides an excellent example of nearly perfect data being generated when the main transition paths and emission wavelengths are combined with their peak areas for calculation.

Another important part of an analytical technique is the correct identification of the electrolytes. Elemental mixtures within biological tissues make the resulting LIBS spectra complex and make identification of the correct peak origins difficult. The use of element-specific reference samples provides a simple method to overcome this challenge. The complex emission patterns of certain elements can be better investigated under controlled conditions. The complexity of emission lines can be illustrated by the reference spectrum of CaCl_2_ (Fig, 5) in this study. Due to the existence of two gaseous Ca states (Ca^+^ and Ca^++^), the emission lines were distributed over the entire range of the spectrometer. Ca can be bound to proteins, while only 50% of Ca is free-flowing in biological fluids (serum, cells, etc.)^42^. In addition, crystallization during the air-drying process of the filter paper may result in CaCl_2_ crystals containing two hydrate water molecules, leading to the suppression of emission lines in those samples. Consequently, when interpreting the emission line pattern of a particular element, the individual interactions of that element must be considered.

Linearity and proper understanding of complex emission line spectra are important for LIBS as an analytical tool and for usage in clinical biomarker studies. However, robustness and feasibility in real samples are even more critical for an analytical assay^21,43^. To evaluate the clinical significance of the results of the current study, the calculation of Lorentzian peak shape areas and the combination of major peak areas of given elements were applied to real human tissue samples. As previously described, the LIBS signal is strongly influenced by the consistency, moisture, and density of the sample^9,10,12,13,19,44^. Notwithstanding these difficulties, clinical biomarkers must be able to accurately detect even miniscule differences. Unfortunately, natural biological variability, together with analytical variability, often leads to poor results. Therefore, in most studies, it is challenging to efficiently separate groups and reliably compare low-intensity peaks with high-intensity peaks. The results of the current study (Fig. 1) are consistent with assessments of other studies^24,25^, showing that the biological significance of small peaks tends to be overestimated, while that of large peaks tends to be underestimated.

To overcome these hurdles, correct quantitative measurement is key. For human tissue samples, almost all measured emission lights and wavelengths could be assigned to their elements of origin. Simple calibration curves, as established in other quantitative analysis methods, such as HPLC–MS, also show how much information can be generated in this way for LIBS experiments. However, the measurements in this study must be evaluated in light of the fact that the tissue was examined ex vivo and not immediately after its collection. In contrast to animal experiments performed immediately after the animals are killed^19,44^, tissue degeneration, protein degradation, desiccation, and osmotic processes have an unmeasurable effect on electrolyte concentrations and on the spectra obtained in this study. Due to its natural inhomogeneity, low tissue density, and high water content, muscle tissue was deliberately chosen as a challenging sample through which to validate the approach proposed in this study. Since this work predominantly focused on the detailed derivation of the underlying principles of spectra evaluation, validation of the approach was limited to muscle tissues from 2 randomly selected patients.

In combination with multivariate statistics, soft tissues with high similarity can be distinguished using LIBS^45^. In the muscle cells of patient A, Ba and a small amount of Sr, in addition to Ca (Figs. 3 and 4), were detected. This is interesting because Ba is rarely discovered in human physiology. In addition to a relatively high Ba concentration, which measured 5–20% higher than the Ca content, approximately 0.5–1% Sr was found in the muscle sample of patient A. This made sense because Ba and Sr often occur together and chemically belong to the alkaline earth group. For the first time, LIBS made it possible to detect Ba and Sr in human cells. It is well known that Ba ions initially migrate into muscle cells^46^ and, at higher concentrations, weaken muscle strength^47^. At the concentrations determined, Ba is not toxic in any way^48^. The origins of Ba and Sr are usually plant-based; however, both elements can also be found in drinking water. Sr has been detected in the bones of Ancient Rome gladiators, attributed to a vegan diet or consumption of ascetic drinks^49^. This example demonstrates the ease with which exotic elements in human tissues with LIBS can be detected. In this context, LIBS may represent an exciting alternative to elaborate analytical methods for forensic investigations^50^.

The use of electrolyte ratios allows for comparisons to be made between different analytical methods and biological compartments, as well as LIBS signal variability. Although the use of a ratio is similar to data normalization, widely scattered results with low single-shot sensitivity have been reported when electrolyte ratios were calculated based on peak intensities only^51,52^. In this study, after calculating the Lorentzian peak shape areas and combining the major peak areas of the respective elements, Na/K ratios with low standard deviations (shown in Table 1) were obtained. This demonstrates that good reproducibility and robustness can be achieved even with native tissue samples. For the biological interpretation of the calculated ratios, the relationship between the laser spot diameter (240 μm) and the average muscle cell size (20–40 μm) must be considered. The measured LIBS Na/K ratio of the muscle tissue shows that the laser analyzes mainly human muscle cells because the ratios are very small and close to the theoretical electrolyte cell concentration^53^. In this context, a large laser spot diameter was deliberately chosen to obtain a better average value over the plasma conditions.

To the authors’ knowledge, this study demonstrates for the first time that calculating Lorentzian peak shape areas and combining the major peak areas of given elements can reduce shot-to-shot variance in natively lasered human biomaterial. With this in mind, the results of this study must be validated in a larger number of cases and applied to other natively lasered tissue types. Hyphenated systems like LIBS-Raman spectroscopy^7,8^, coherent anti-Stokes spectroscopy^35,36^ or multi-modal optical spectroscopic sensors^54,55^ can certainly analyze molecular and elemental behavior within biological tissues in even greater detail and with higher spatial resolution. This work has focused on LIBS in particular, as LIBS is not limited to surface analysis, but can also function under difficult analytical conditions, such as living or native tissue cells. Therefore, the biological significance of peak area calculation can serve LIBS-based systems in the future when the electrolyte balance of cells will be analyzed.

## Conclusion

The LIBS spectra investigated in this work show a wealth of detail. Most of the emission lines could be identified with pure salt reference samples. Due to the high linearity of the KCl calibration curve and the low standard deviation of the Na/K ratio in the human muscle tissue samples, the present study demonstrates that consideration of peak shape transition pathways not only provides reproducible results under the condition of homogeneous samples but can also be applied to native human biomaterial. Regarding our evaluation, linearity, specificity, and robustness were successfully demonstrated for the proposed analytical method of LIBS spectra in human biomaterial.

Direct visualization of cell-level processes using LIBS spectra can contribute to a better understanding of physiological and pathological processes in different scenarios. Real-time examination of tissue samples supported by robust evaluation methods can help to visualize minimal differences in clinical biomarker studies. The results of the present study could expand the range of indications for laser systems and increase confidence in the safety of their application in oncological diagnostics and surgery. Future trends in bio-applied LIBS techniques could focus on in situ applications^38^ and elemental imaging procedures^14,56,57^.

## Data Availability

The datasets generated during and analyzed during the current study are available from the corresponding author on reasonable request.
